# Headache yesterday in Karnataka state, India: prevalence, impact and cost

**DOI:** 10.1186/s10194-016-0669-y

**Published:** 2016-08-25

**Authors:** Timothy J. Steiner, Girish N. Rao, Girish B. Kulkarni, Gopalkrishna Gururaj, Lars J. Stovner

**Affiliations:** 1 Department of Neuroscience, Norwegian University of Science and Technology (NTNU), Edvard Griegs Gate, Trondheim, NO-7491 Norway; 2 Division of Brain Sciences, Imperial College London, London, UK; 3 Department of Epidemiology, National Institute of Mental Health and Neuro Sciences (NIMHANS), Bengaluru, India; 4 Department of Neurology, National Institute of Mental Health and Neuro Sciences (NIMHANS), Bengaluru, India; 5 Norwegian Advisory Unit on Headache, Nevrosenteret Øst, St Olavs University Hospital, Trondheim, Norway

**Keywords:** Headache yesterday, Migraine, Tension-type headache, Medication-overuse headache, Burden, Cost, Health policy, India, Global Campaign against Headache

## Abstract

**Background:**

The Global Campaign against Headache has pioneered evaluation of the prevalence and impact of headache on the preceding day (“headache yesterday”) as a new approach to the estimation of headache-attributed burden, avoiding recall error. We report its application in Karnataka State, southern India.

**Methods:**

In a door-to-door survey, biologically unrelated adults (aged 18–65 years) were randomly sampled from urban and rural areas in and around Bengaluru and interviewed by trained researchers using a validated, structured questionnaire. Enquiry into headache applied ICHD-II diagnostic criteria and included questions about headache on the day preceding the interview (headache yesterday [HY]).

**Results:**

There were 2329 participants (participation proportion 92.6 %; males 1141 [49.0 %], females 1188 [51.0 %]; mean age 38.0 [±12.7] years; 1103 [47.4 %] from rural areas, 1226 [52.6 %] urban). HY was reported by 138 participants (males 33 [2.9 %], females 105 [8.8 %]): the 1-day prevalence of headache was 5.9 %. Mean duration of HY was 7.0 ± 8.5 h, so that 1.7 % of the population (5.9 % * 7.0/24), on average, had headache at any moment in time yesterday. Mean intensity on a scale of 1–3 was 2.0 [±0.8]. Lost productivity due to HY was reported by 83.3 % of participants with HY: 37.7 % able to do less than half of what they had planned and 13.0 % able to do nothing. Productivity loss at population level (being the productivity loss within the entire adult population, every single day, attributable to headache) was 3.0 %.

**Conclusions:**

This method of enquiry, free from recall error, confirmed a very high level of headache-attributed burden in Karnataka: previous estimates based on 3-month recall may even have been too low. Until another study is done in the country, these are the best data for all India. They demonstrate need for action nationwide to mitigate this burden, and correct action will ultimately almost certainly be cost-saving.

## Background

The very heavy disability burden attributable to headache disorders is becoming increasingly clear [1, 2]. The Global Burden of Disease Study 2010 (GBD2010) ranked tension-type headache (TTH) and migraine as the second and third most common diseases worldwide [3], but many of the data included in this world survey derived from heterogeneous studies performed with varying methods over a period of nearly 30 years. In all regions except the Far East, more recent studies–of migraine in particular–have generally yielded higher prevalence estimates than the global mean estimates of GBD2010 [3] or GBD2013 [1]. This has been particularly apparent in low- and lower-middle-income countries: Georgia [4], India [5], Nepal [6], Zambia [7], Ethiopia (unpublished) and Pakistan (unpublished). In part this is explained by the careful methodology of recent studies [8, 9]. To a greater extent it may be because many earlier published studies excluded probable migraine: many reports are silent on how ICHD diagnostic criteria were applied with respect to definite and probable migraine; some explicitly excluded the latter, but a larger number may have done so without making this clear.

The expectation, as the Global Campaign against Headache [10, 11] continues its drive to conduct population-based studies in those parts of the world where data are currently lacking–mostly in low- and lower-middle-income countries, is that global mean prevalence estimates will continue to rise. With them, estimates of burden will rise also, as they have done through the iterative GBD studies since 2000 [2]. These estimates are the basis of needs assessments informing health policy and allocation of health-care resources.

When disease burdens are large and the call upon resources is substantial, estimates need to be as accurate as possible. The methodology for measuring headache-attributed burden is a relatively new and still developing science [8, 9]: in the last 30 years, many population-based studies of headache have assessed prevalence only. It is a field of innovation to which *Lifting The Burden* (LTB) [12, 13] has actively contributed in leading the Global Campaign against Headache. The components of headache-attributed burden are wide-ranging, and some are challenging to measure [9]. Symptom burden can be measured in terms of frequency, intensity and duration. The GBD studies use frequency and duration to estimate the proportion of time spent with symptoms, and apply a disability weight to this proportion of time to calculate the associated health loss [3]. For socioeconomic impact, the Headache-Attributed Lost Time (HALT) Index [14] enquires into effect of headache on productivity. In its original form (HALT-90), it relied upon a respondent’s recall over the 3 months prior to the interview, that period of enquiry being intended to gather a representative view of each participant’s lost productive time. This approach has its uses, but is subject to the limitations, errors and possible biases of recall over such a long period.

LTB has pioneered an alternative approach to avoid the problems associated with recall error, which is enquiry into headache occurring on the day before the interview (“headache yesterday”; HY) [8]. Recollection of HY, its symptoms and whatever impact they may have had, is likely to be highly reliable. While what happened yesterday is not representative, or therefore indicative, of an individual’s headache-attributed burden over longer periods of time, at population level it is. This approach does, however, require a large number of participants, since far fewer people have headache in 1 day than in 3 months.

A question subset concerning HY [9] has been included in several recent large epidemiological studies supported by LTB within the Global Campaign against Headache: in China [15], Russia [16], countries of the Eurolight project [17] and several others not yet published. Here we report the analysis of HY in Karnataka State, southern India, and make comparisons with estimates based on 3 months’ recall [18]. We apply the analysis in making an estimate of socioeconomic burden attributable to headache disorders in Karnataka State, and consider extrapolating this to all India.

## Methods

The methods of the study have been published in detail previously [19] and are described only briefly here.

### Study design and sampling

This was a cross-sectional survey, conducted during May to November 2009. It sampled urban and rural areas in and around Bengaluru: Kempegowdanagara, an urban administrative ward in the city of Bengaluru, and Uyamballi and Doddaaladahalli, two large villages located 75–80 Km from Bengaluru. Trained interviewers selected households randomly through multistage cluster sampling. They called at each household unannounced, listed all adult members (aged 18–65 years), randomly selected one and interviewed that person.

### Enquiry and diagnosis

The survey instrument was a cultural adaptation of LTB’s structured HARDSHIP questionnaire [9], translated into the local language (Kannada) in accordance with LTB’s translation protocol for hybrid documents [20], and validated [19]. It began with demographic enquiry. The headache screening question (“Have you had headache during the last year?”) and diagnostic questions based on ICHD-II [21] were followed, for those reporting headache, by multiple question sets enquiring into various aspects of burden, including the HALT questionnaire [14]. Participants reporting more than one headache type were asked to focus only on the one that was subjectively the most bothersome for purposes of diagnosis and burden attribution.

Diagnoses were made algorithmically. Participants with headache on ≥15 days/month were first identified; among these, any with medication overuse were diagnosed as probable MOH (pMOH), the remainder as “other headache on ≥15 days/month”. The algorithm then applied ICHD-II criteria to all other cases in hierarchical sequence: first for definite migraine, then for definite TTH, then for probable migraine and finally for probable TTH. Remaining cases were considered unclassifiable. In the analysis, definite migraine and probable migraine were grouped as migraine, and definite TTH and probable TTH as TTH.

Questions on HY (in response to “Did you have headache yesterday?”) included duration of HY (in hours up to 24), intensity of HY (reported by participants on a three-point categorical score [“not bad”, “quite bad” and “very bad”]) and usage of acute medications. Lost productivity because of HY was reported as what had been done yesterday considered with regard to what had been planned or expected to be done: “everything”, “more than half “, “less than half” or “nothing”. The analysis of HALT offsets those who had done less than half against those who had done more than half, counting the former as doing nothing and the latter as doing everything [9, 14].

### Statistics and analyses

Data were entered into a secure database and statistical analyses were performed using EPI INFO [22] and SPSS version 15.

We used proportions (%) to report prevalences, with 95 % confidence intervals (CIs) when appropriate. We used means, with standard deviations (SDs) and/or standard errors (SEs), and medians to summarise the distributions of continuous variables. Headache intensity responses “not bad”, “quite bad” and “very bad” were equated to “mild”, “moderate” and “severe” and converted to a numerical rating scale 1–3, which we treated as continuous data. We used Fisher’s exact test for significance of differences. We set the level of significance at *p* < 0.05.

We calculated the predicted 1-day prevalence of any headache and of headache types from the 1-year prevalence and estimated mean frequency (F) in days/year (extrapolated from the last 3 months) using the formula:

$$ 1-\mathrm{day}\ \mathrm{prevalence}=1-\mathrm{year}\ \mathrm{prevalence}\ast \mathrm{F}/365 $$

We calculated total lost productivity as a proportion (%), the numerator being the sum of those reporting nothing or less than half done and the denominator the number (N) who reported HY.

## Results

There were 2329 participants (1141 [49.0 %] male, 1188 [51.0 %] female; mean age 38.0 [±12.7] years; 1103 [47.4 %] from rural areas, 1226 [52.6 %] urban). The participation proportion was 92.6 % (eligible population *n* = 2514). The distributions of gender, age and habitation in the participating sample have been described in detail previously and were comparable to those of the population of Karnataka [19]. As reported previously, the 1-year prevalence of any headache was 63.9 %; the age-standardised 1-year prevalence of migraine was 25.2 % and of TTH was 35.1 %; the age-standardised prevalence of all causes of headache on ≥15 days/month was 3.0 % and of pMOH was 1.2 % [5].

### Headache yesterday

HY was reported by 138 participants, representing a 1-day prevalence of headache of 5.9 %. The predicted 1-day prevalence of any headache calculated from its 1-year prevalence of 63.9 % and estimated mean frequency of 32.0 days/year [5] was 5.6 %, a very close match.

HY was reported by over three times as many females (8.8 %) as males (2.9 %) (Table 1). Reporting of HY varied with age, but with wide and overlapping confidence intervals. Almost three quarters of HY (73.9 %) was reported by rural participants, despite that they made up less than half (47.4 %) of the participating sample. These data are also shown in Table 1.

**Table 1:** Characteristics of participants reporting headache yesterday (HY) and population prevalence of HY by group (*N* = 2329)

Characteristic	Reported cases of HY in defined group n (%)	N in defined group	Population prevalence of HY in defined group (%) [95 % CI]
All cases of HY	138 (100.0)	2329	5.9 [5.0–7.0]
Gender			
Male	33 (24.0)	1141	2.9 [2.1–4.0]
Female	105 (76.0)	1188	8.8 [7.4–10.6]
Age (years)			
<25	18 (13.0)	353	5.1 [3.3–7.9]
25 to 34	35 (25.4)	627	5.6 [4.0–7.7]
35 to 44	41 (29.7)	603	6.8 [5.1–9.1]
45 to 54	23 (16.7)	409	5.6 [3.8–8.3]
55 to 65	21 (15.2)	337	6.2 [4.1–9.3]
Habitat			
Rural	102 (73.9)	1103	9.3 [7.7–11.1]
Urban	36 (26.1)	1226	2.9 [2.1–4.0]

HY was reported to be typical of their usual most bothersome (diagnosed) headache by only 95 (68.8 %) of those with HY. This limited the inferences we could draw about HY diagnostically. Among these 95 only, we could make reasonable assumptions as to diagnosis; on these, we based calculations of predicted 1-day prevalences by headache type (Table 2). There was an exact match for migraine and a close match for pMOH; TTH and other headache on ≥15 days/month were underreported yesterday, and may have been preferentially among the cases of HY not considered typical of the usual most bothersome headache. For all HY, as noted above, there was a good match.

**Table 2:** Prevalence of headache yesterday according to diagnosis (when recognized as most bothersome headache), and comparison with predicted 1-day prevalence based on reported frequency and 1-year prevalence

Headache type	Headache yesterday: with diagnosis (*N* = 95) or all cases (*N* = 138)		Predicted 1-day prevalence		
	n (%)	Observed 1-day prevalence (%) (*N* = 2329)	1-year prevalence ^a^ (%)	Mean headache days/year	Calculated 1-day prevalence (%)
Migraine	44/95 (46.3 %)	1.9	25.2	28 ^b^	1.9
Tension-type headache	23/95 (24.2 %)	1.0	35.1	17 ^b^	1.6
Probable medication-overuse headache	13/95 (13.7 %)	0.6	1.2	226 ^b^	0.7
Other headache on ≥15 days/month	15/95 (15.8 %)	0.6	1.8	259 ^b^	1.3
Unknown headache yesterday	43/138	1.8	-	-	-
All headache yesterday	138	5.9	63.9	32.0 ^a^	5.6 ^a^

^a^From reference [5]

^b^From reference [18]

Mean duration of HY was 7.0 ± 8.5 h. From this we calculated that 1.7 % of the population (5.9 % * 7.0/24), on average, had headache at any moment in time yesterday. The median was 3 h; therefore HY was quite short-lasting in more than half of cases. If this reflected usage of acute medications, these were taken by 46.3 % of participants with HY–that is, 2.5 % of the total survey sample. By implication this meant that, on any day, 2.5 % of the adult population were using acute medication for headache. A further 36.9 % used local applications of various sorts, including 4.3 % who used both medication and local applications. Headache intensity was severe in 29.7 %, moderate in 46.4 % and mild in 23.9 %. Mean intensity on a scale of 1–3 was therefore 2.0 [±0.8] (SE: 0.07).

Lost productivity due to HY was reported to some degree by 83.3 % of participants with HY: 37.7 % had been able to do less than half of what they had planned and 13.0 % could do nothing. Applying the concept behind the analysis of HALT (see Methods [14]), we found that HY cost those affected by HY half (50.7 %) of their productivity yesterday. We looked at this by diagnosis in those for whom we had diagnostic information (Table 3). This analysis indicated that migraine cost much more lost productivity than TTH (*p* = 0.048 [Fisher’s exact, 2-tailed]), while pMOH eclipsed even migraine (but with small numbers, *p* = 0.75). To calculate the productivity loss due to HY at population level we multiplied 50.7 % by the overall prevalence of HY of 5.9 %; this gave an estimate of 3.0 % as the productivity loss within the entire adult population, every single day, attributable to headache.

**Table 3:** Lost productivity because of headache yesterday according to diagnosis (when recognized as most bothersome headache)

Amount of lost productivity	Headache yesterday with diagnosis (*N* = 95)				Any headache yesterday (*N* = 138)
	Migraine	Tension-type headache	Probable MOH	Other headache on ≥15 days/month	
N reporting headache yesterday (X)	44	23	13	15	138
Could do nothing at all (n) (Y)	6	1	2	2	18
Could do less than half (n) (Z)	20	6	7	3	52
Estimated lost productivity yesterday ([Y + Z]/X) (%)	60.5	30.4	69.2	33.3	50.7

*MOH* medication-overuse headache

## Discussion

The principal finding was a 1-day prevalence of headache of 5.9 %, with the implication that this proportion of the adult population (one in 17) had headache on each and every day. Mean duration of HY was 7.0 ± 8.5 h, so that 1.7 % of the population (5.9 % * 7.0/24), on average, had headache at any moment in time yesterday (and, again by implication, at any moment on any day). Mean intensity on a scale of 1–3 was 2.0. Lost productivity was reported by most (83.3 %) participants with HY, of whom 37.7 % could do less than half of what they had planned and 13.0 % could do nothing. Productivity loss due to HY at population level (being the productivity loss within the entire adult population, every single day, attributable to headache) was 3.0 %.

The large difference in prevalence of HY between rural (9.3 %) and urban populations (2.9) was in part explained by the higher rural 1-year prevalence of headache (71.2 % versus 57.3 %), thought to be due to adverse rural socioeconomic conditions (diet, stress and relative poverty) [5]. In addition, the poor rural availability and utilization of health-care facilities were likely to contribute to more frequent headache and therefore more HY. Probable MOH was more prevalent in rural (1.5 %) than in urban areas (0.9 %) [5]; because of its high frequency, this disorder is a major contributor to HY despite its low prevalence.

It is a limitation of enquiry into HY that diagnosis cannot reliably be based on a single headache episode; unless a respondent is able to assert that HY was typical of a recurrent headache that has been diagnosed, its nature will not be known. In this survey, only two thirds could do this, with the result that our inferences on headache types, as opposed to all headache, are unavoidably constrained. From a public-health perspective this may not be of great importance; the key messages here emphasise the ubiquity of headache, its frequency, its symptom burden, which can be assessed regardless of headache type, and its impact at population level (3.0 % loss of productivity).

This is the fourth published analysis of HY, following those in China [15], eight countries of Europe [17] and Russia [16]. Comparison with Russia is of most interest because headache prevalences in China are much lower [23] and the data from the Eurolight study were not entirely population-based [24]. In Russia, 14.5 % of participants, also aged 18–65 years, reported HY [16]. This much higher proportion (14.5 % versus 5.9 %) reflected a lower prevalence of migraine in Russia than in Karnataka (20.3 % versus 25.2 %) and lower prevalence of TTH (30.9 % versus 35.1 %), but a much higher prevalence of headache on ≥15 days/month (10.5 % versus 3.0 %) [5, 25]. As noted above, high-frequency headache has a dominant influence on HY.

The mean duration of HY of 7 h in Karnataka compared with 6 h in Russia, where there was a higher rate of medication use [16]. While we calculated that 1.7 % of the adult population in Karnataka had headache at any moment in time, in Russia, the proportion was much higher (3.6 % [16])–more than double. However, China reported a very similar 1.8 % [15]. In terms of pain intensity, the mean of 2.0 on a scale of 1–3 was much the same as the 2.1 reported in Russia [16], and expected to be disabling. The consequences were seen in the per-person lost productivity of 50.7 % and population-level lost productivity of 3.0 %. Per-person lost productivity was lower in Russia, at 39.8 %, but, with the higher prevalence, population-level lost productivity attributed to HY was quite a lot higher than in Karnataka, at 5.8 % [16]. Notwithstanding this, the 3.0 % losses in Karnataka represent a major socioeconomic drain.

While 5.9 % of participants reported HY in Karnataka, this number was closely corroborated by the predicted 1-day prevalence of headache of 5.6 %. In all three earlier published studies of HY, similar internal consistency has been seen between reported HY and prediction of 1-day headache prevalence based on estimated 1-year prevalence and recalled frequency during the preceding 3 months [15–17]. What was underestimated in recall over 3 months was the productivity loss, which was calculated at population level in Karnataka at only 1.8 % [18])–seemingly a major error. Similar underestimates in recall were reported in Russia [16].

The crucial question, which has been raised before [5, 18], is whether and to what extent these findings are representative of this culturally and environmentally very diverse country and can, therefore, be extrapolated to all India. The truth is that we do not know: we have drawn attention to the need for at least one further similar study in the country, perhaps in the north, seeking corroboration [18]. In the meantime, we reiterate that these are the best data available for the country. If it is the case that 1.7 % of the Indian adult population (approximately 14 million people), or anything in the region of this number, have disabling headache at every moment, this signals a pressing need to do something about it: cost-effective treatments are available [26]. At the least, the further study we have called for should be commissioned urgently. The do-nothing option is not cost-free: it incurs the estimated 3.0 % lost productivity, which is likely to be reflected in a correspondingly diminished gross domestic product. It should be noted that this survey was conducted among the population aged 18–65 years: ie, those of working age, among whom productivity losses have most impact on the population economy. The cost is therefore huge.

The strengths of our study were several. Enquiry into headache yesterday is a powerful approach to population-based burden-of-headache studies, virtually eliminating recall error and any bias that may follow from this [9]. Our sample was large and representative, and the non-participation proportion was low (7.4 %) [5, 19]. Through cold-calling and face-to-face interviews, we avoided the previously described bias of “headache today” [17]: participants with headache on the day they receive a questionnaire by post may defer responding until the first day of headache-freedom, and then spuriously report HY.

## Conclusions

Using HY as a method of enquiry free from recall error, we have confirmed a very high level of headache-attributed burden in Karnataka. Previous estimates based on 3-month recall [18] may even have been too low. Until another study is done in the country, these are the best data for all India. We repeat our call for action, reiterating that correct action will ultimately almost certainly be cost-saving [27].
